# Smart Sound Processing for Defect Sizing in Pipelines Using EMAT Actuator Based Multi-Frequency Lamb Waves

**DOI:** 10.3390/s18030802

**Published:** 2018-03-07

**Authors:** Joaquín García-Gómez, Roberto Gil-Pita, Manuel Rosa-Zurera, Antonio Romero-Camacho, Jesús Antonio Jiménez-Garrido, Víctor García-Benavides

**Affiliations:** 1 Department of Signal Theory and Communications, University of Alcalá, Ctra. Madrid-Barcelona, km. 33,600, 28805 Alcalá de Henares, Spain; joaquin.garciagomez@uah.es (J.G.-G.); manuel.rosa@uah.es (M.R.-Z.); 2 Innerspec Technologies Europe S.L, Av. de Madrid 2, 28802 Alcalá de Henares, Spain; aromero@innerspec.com (A.R.-C.); jjimenez@innerspec.com (J.A.J.-G.); vgarcia@innerspec.com (V.G.-B.)

**Keywords:** EMAT actuators, Lamb waves, pipeline inspection, defect sizing, smart sound processing

## Abstract

Pipeline inspection is a topic of particular interest to the companies. Especially important is the defect sizing, which allows them to avoid subsequent costly repairs in their equipment. A solution for this issue is using ultrasonic waves sensed through Electro-Magnetic Acoustic Transducer (EMAT) actuators. The main advantage of this technology is the absence of the need to have direct contact with the surface of the material under investigation, which must be a conductive one. Specifically interesting is the meander-line-coil based Lamb wave generation, since the directivity of the waves allows a study based in the circumferential wrap-around received signal. However, the variety of defect sizes changes the behavior of the signal when it passes through the pipeline. Because of that, it is necessary to apply advanced techniques based on Smart Sound Processing (SSP). These methods involve extracting useful information from the signals sensed with EMAT at different frequencies to obtain nonlinear estimations of the depth of the defect, and to select the features that better estimate the profile of the pipeline. The proposed technique has been tested using both simulated and real signals in steel pipelines, obtaining good results in terms of Root Mean Square Error (RMSE).

## 1. Introduction

Ultrasonic techniques have demonstrated over the years to be really useful for Non-Destructive Testing (NDT) examinations [1,2,3]. Conventional ultrasounds are primarily generated taking advantage of the piezoelectric effect. Although it is an efficient way of generating ultrasounds, a proper coupling between the transducer and test specimens is needed, which is a disadvantage. Therefore, materials inspected by conventional ultrasounds are covered with a thin layer of fluid. EMAT (Electro-Magnetic Acoustic Transducer) actuators are able to generate and receive ultrasonic waves without the need to have thorough contact with the surface of the material under investigation [4]. This technology is capable of generating multiple types of waves: Lamb, shear, longitudinal and Rayleigh. Besides, when EMAT technique is implemented with a meander-line-coil, the waves are generated in a directional way [5,6]. This fact is interesting since it allows differentiating between circumferential and axial scans.

A highlighted application of this technology is the pipeline inspection [7]. On the one hand, some manuscripts have focused on the defect detection and location in its circumferential path, mainly using shear waves [8,9,10]. However, it is important to obtain not only the position of the defect, but also its residual thickness. For the companies it is interesting to know this parameter, since it is a vital factor to make the decision to replace a section of the pipeline [11]. On the other hand, there have been some proposals of sizing techniques applied to pipeline inspection, mainly based on the analysis of the physical mode [12], but the distortion caused by the defects over the different modes strongly varies with the shape of the defect [12,13,14]. Smooth defects usually reflect less energy than abrupt ones, independently of the residual thickness of the pipe caused by the defect. Thus, the amplitude of the received echo is strongly related not only to the depth of the defect but also to its hardness, and both the amplitude and the time of arrival of the wrap-around signal vary with the length of the defect.

In general, the studies have followed the same line with regard to the information extracted from the wave modes. The most relevant and used parameters are the amplitude and the phase from the received signal [14,15]. Once this information is obtained, the use of Smart Sound Processing (SSP) techniques is suitable for solving sizing problems using EMAT guided waves. These methods involve extracting useful information from the sensed acoustic signals and applying nonlinear techniques to obtain estimations of useful parameters. Other proposals have applied this type of techniques in their studies, including: Artificial Neural Networks [16], Neural Networks with large number of neurons [17] or Adaptive Neuro-Fuzzy Inference Systems [18]. Using this type of methods is interesting to excite the coil at multiple frequencies, as the behavior of the Lamb modes is different depending on this parameter, and SSP allows to combine all this information and get better defect estimation results.

In this sense, this paper studies pipeline inspection mixing both the EMAT and SSP techniques. Specifically, EMAT-based Lamb waves will be generated at multiple frequencies. Axial scans will be developed and the circumferential path followed by waves will allow the analysis of the wrap-around signals received. These ultrasound signals will be related to the behavior of the pipeline depending on its profile, conditions and damage. Once these signals are measured, it will be possible to apply SSP techniques in order to get useful information from the amplitude and phase of the multi-frequency signals. In particular, feature selection techniques and Neural Networks-based estimators will be applied. Following this process, it will be feasible to obtain an approximated characterization of the residual thickness along the pipeline.

## 2. Materials and Methods

In this section the sensorization of Lamb waves through EMAT actuators will be described. Initially, EMAT technology and the fundamentals of Lamb waves will be introduced. A brief hardware description will be made at the end of the section as well.

### 2.1. Lamb Wave Generation Using EMAT Actuators

EMAT transducers consist of a coil wire and a magnet. The alternating electrical current flowing through the coil wire placed in a uniform magnetic field (*B*) near the surface of a ferromagnetic material, induces surfaces currents (Eddy Currents, *J*) in the material. The field generated by electrical coils interacts with the field generated by the magnet producing a Lorentz force (*F*) according to Equation (1).

(1) F=J×B

**Figure 1:**
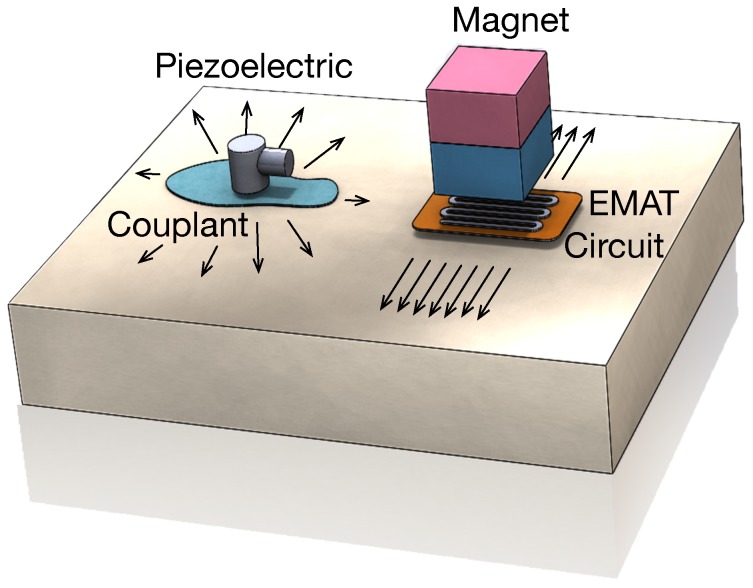
Conventional Ultrasound vs EMAT.

The disturbance is applied to the lattice of the material, producing an elastic wave. In a reciprocal process (reception of an ultrasonic wave), the interaction of elastic waves in the presence of a magnetic field induces currents in the EMAT receiver coil circuit. In Figure 1 a comparative between the generation of ultrasonic waves using conventional ultrasound methods and using EMAT technology is represented.

The advantages of using EMAT over piezoelectric transducers are: as the transduction process occurs within an electromagnetic depth skin, it is a couplant free technique; it is insensitive to surface conditions, being capable of inspecting rough, dirty (oily/wet), oxidized or uneven surfaces; inspection can be carried out on flat, curved or complex surfaces; it allows high speed inspections (up to 60 m/s), high temperature inspections and low temperature inspections, and it can generate Lamb, Shear Horizontal (SH), Shear Vertical (SV), Longitudinal and Rayleigh waves due to its good selectivity in frequency. On the other hand, the challenges of EMAT are the high level of power required, the bigger size of the transducers and the lower Signal to Noise Ratio (SNR). Besides, the material under inspection needs to be conductive.

Guided Wave Testing is a NDT technique that employs ultrasonic stress waves that propagate along a structure while guided by its boundaries. Guided waves permit covering long distances from a single point with a limited number of sensors, being very effective for rapid scanning of pipelines and tanks. On relatively thin structures, it is possible to generate volumetric guided waves that fill up the material and permit a complete, volumetric inspection. The most common types of volumetric waves are SH and Lamb.

Lamb waves travel throughout the material with both vertical and forward motion in an elliptical pattern. These waves are dispersive by nature, and very sensitive to thickness variations. They can be classified in symmetric (also known as longitudinal) and asymmetric (also known as flexural) modes. The introduction of boundary conditions makes Lamb wave problems inherently more difficult than the more conventional bulk waves. Unlike the finite number of modes present in a bulk wave problem, there are an infinite number of modes associated with a given Lamb wave application. That is, a finite body can support an infinite number of different Lamb wave modes. Now the generation of the Lamb wave modes will be described. With this purpose, Lamé parameters will be defined. Lamé parameters are two material-dependent quantities denoted by λ (Lamé’s first parameter) and μ (Lamé’s second parameter). They are defined by Equations (2) and (3).

(2) $$\lambda =\frac{E\nu }{\left(1+\nu )(1-2\nu \right)}$$

(3) $$\mu =\frac{E}{2(1+\nu )}$$

where *E* is the Young’s modulus, which measures the stiffness of a material, and ν is the Poisson’s ratio, which is an elastic constant that measures how an elastic, linear and isotropic material is narrowed when it is longitudinally stretched.

Then the elastic wave equation needs to be taken into account.

(4) $$\mu \nabla^{2}u+\left(\lambda +\mu \right)\nabla \nabla \cdot u=\rho \left(\frac{\partial^{2}u}{\partial t^{2}}\right),$$

where ρ represents the density of the material under inspection. Applying the Helmholtz decomposition, the displacement field u can be split into a rotational component ∇xH and an irrotational component ∇ϕ:

(5) u=∇ϕ+∇xH

Then, the system of partial differential equations can be rewritten as:

(6) $$c_{L}\nabla^{2}\phi =\frac{\partial^{2}\phi }{\partial t^{2}}$$

(7) $$c_{T}\nabla^{2}H=\frac{\partial^{2}H}{\partial t^{2}},$$

where $c_{L}=\sqrt{(\lambda +2\mu )/\rho }$ and $c_{T}=\sqrt{\mu /\rho }$ represent the sound velocity for the longitudinal and transversal modes, respectively.

To continue with the analysis, an infinitely plate extended in the *x* and *y* directions will be assumed. Furthermore, it is considered that the wave propagates in the *x* direction, the fields are uniform in the *y* direction and boundary conditions at z=−h/2 and z=+h/2, where *h* is the thickness of the plate, are considered traction free.

Assuming that the particle displacement is zero in the *y* direction ($u_{y}=0$) and the only rotation is about the *y* axis ($H_{x}=H_{z}=0$) in Equation (4), the Lamb wave equations are obtained [19,20].

(8) $$\mu \left(\frac{\partial^{2}}{\partial x^{2}}+\frac{\partial^{2}}{\partial z^{2}}\right)u_{x}+\left(\lambda +\mu \right)\frac{\partial }{\partial x}\left(\frac{\partial u_{x}}{\partial x}+\frac{\partial u_{z}}{\partial z}\right)=\rho \left(\frac{\partial^{2}u_{x}}{\partial t^{2}}\right)$$

(9) $$\mu \left(\frac{\partial^{2}}{\partial x^{2}}+\frac{\partial^{2}}{\partial z^{2}}\right)u_{z}+\left(\lambda +\mu \right)\frac{\partial }{\partial z}\left(\frac{\partial u_{x}}{\partial x}+\frac{\partial u_{z}}{\partial z}\right)=\rho \left(\frac{\partial^{2}u_{z}}{\partial t^{2}}\right)$$

Applying the restriction in the frontiers:

(10) $${\left.\mu \left(\frac{\partial u_{x}}{\partial z}+\frac{\partial u_{z}}{\partial x}\right)\right|}_{z=\pm \frac{h}{2}}=0$$

(11) $${\left.\lambda \frac{\partial u_{x}}{\partial x}+\left(\lambda +2\mu \right)\frac{\partial u_{z}}{\partial z}\right|}_{z=\pm \frac{h}{2}}=0$$

Focusing on Lamb waves, they are composed of two waves (one longitudinal and one transversal) traveling at different angles $\theta_{L}$ and $\theta_{T}$, where the first one represents the longitudinal angle and the second one the transversal one. Therefore, the wave number *k* is related to the component of the waves that propagates in the *x* direction at velocity $c_{p}$:

(12) $$k_{L}\cos\theta_{L}=k_{T}\cos\theta_{T}=k=\frac{2\pi f}{c_{p}}=\frac{\omega }{c_{p}},$$

where $k_{L}=2\pi f/c_{L}$, $k_{T}=2\pi f/c_{T}$, ω is the angular velocity, $c_{L}$ is the longitudinal component of the velocity and $c_{T}$ is the transversal component of the velocity.

On the other hand, the displacement of each independent wave in the *z* axis can be obtained using Equations (13) and (14).

(13) $$\alpha_{L}=k_{L}\sin\theta_{L}=k_{L}\sqrt{1-\cos^{2}\theta_{L}}=\sqrt{k_{L}^{2}-k^{2}}=\sqrt{\frac{\omega^{2}}{c_{L}^{2}}-k^{2}}=\omega \sqrt{\frac{1}{c_{L}^{2}}-\frac{1}{c_{p}^{2}}}$$

(14) $$\alpha_{T}=k_{T}\sin\theta_{T}=k_{T}\sqrt{1-\cos^{2}\theta_{T}}=\sqrt{k_{T}^{2}-k^{2}}=\sqrt{\frac{\omega^{2}}{c_{T}^{2}}-k^{2}}=\omega \sqrt{\frac{1}{c_{T}^{2}}-\frac{1}{c_{p}^{2}}}$$

where $\alpha_{L}$ and $\alpha_{T}$ represent the longitudinal and transversal displacements of the wave, and $\theta_{L}$ and $\theta_{T}$ are the angles related to these displacements. Taking into account that the wave is reflected in the surfaces, applying the boundary conditions and simplifying the equations, the dispersion equation of the Lamb modes is obtained. Equation (15) refers to the symmetric modes and Equation (16) refers to the asymmetric ones.

(15) $$4k^{2}\alpha_{L}\alpha_{T}\sin\left(\frac{\alpha_{L}h}{2}\right)\cos\left(\frac{\alpha_{L}h}{2}\right)+\sin\left(\frac{\alpha_{L}h}{2}\right)\cos\left(\frac{\alpha_{L}h}{2}\right){\left(\alpha_{T}^{2}-k^{2}\right)}^{2}=0$$

(16) $$4k^{2}\alpha_{L}\alpha_{T}\cos\left(\frac{\alpha_{L}h}{2}\right)\sin\left(\frac{\alpha_{L}h}{2}\right)+\cos\left(\frac{\alpha_{L}h}{2}\right)\sin\left(\frac{\alpha_{L}h}{2}\right){\left(\alpha_{T}^{2}-k^{2}\right)}^{2}=0$$

From the previous equations, it can be figured out that there exists a relation between the excited frequency *f*, the thickness of the pipe *z* and the phase velocity $c_{p}$. More specifically, each mode will move at different $c_{p}$ depending on the other above-mentioned parameters. A similar relation can be obtained using the group velocity $c_{g}$, which is defined in Equation (17).

(17) $$c_{g}=\frac{\partial \omega }{\partial k}$$

**Figure 2:**
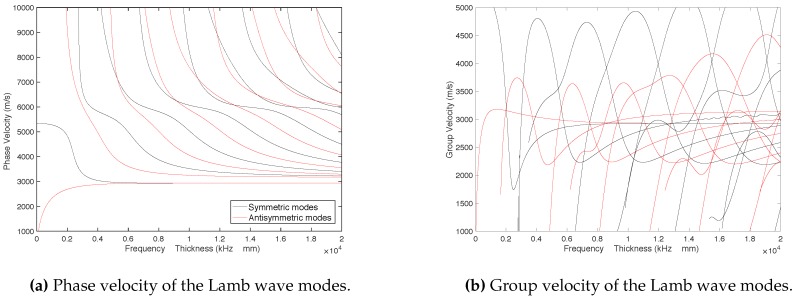
Phase velocity and group velocity depending on the product frequency by thickness.

Graphs showed in Figure 2 were obtained by means of the previous equations for different values of the product frequency by thickness, where phase velocity is represented in Figure 2a and group velocity in Figure 2b. As it can be observed, the relation between the velocities and the frequency is non-linear, so there exists dispersion in the Lamb wave propagation.

In Figure 2, black lines increasingly represent symmetric modes from left to right (S0, S1, S2...), while red lines represent in the same way antisymmetric modes (A0, A1, A2...). These graphs correspond to a steel pipe with the following parameters: Young’s modulus $E=200\cdot 10^{9}$ N/m${}^{2}$, Poisson’s ratio ν=0.3 and density ρ=7700 kg/m${}^{3}$.

Now the methodology followed to generate and receive signals in the pipeline will be described. The transducer consists on a meander-line-coil which generates two signals per loop in the test piece (one per meander). These waves will be characterized by the wavelength which depends on the separation of the meanders.

The following equations are valid for one mode and then the same procedure will be applied iteratively for all the modes which appear at a set frequency. Thus, wave equation is set depending on the group and phase velocities. Considering *f* as the excited frequency, the transmitted signal s(x,t) will be generated according to Equation (18).

(18) $$s\left(x,t\right)=\sin\left(2\pi f\left(\frac{x}{c_{p}}\pm t\right)+\phi \right),$$

where ϕ is a phase term that controls the phase of the transmitted signal.

In a real case, the transmitted signal includes an envelope w(t) that generates the transmitted wave packet p(x,t). This envelope limits the transmission time, and allows controlling the length of the transmitted pulse. Typically, the length of this envelope is described in function of *C*, the number of cycles included in the wave packet. That is, the length of w(t) will be C/f, where 1/f is the time period corresponding to the excited frequency. This envelope will travel at an average velocity of $c_{g}$, and in general its shape will change with the distance due to dispersion effects.

The inclusion of the time envelope w(t) in the transmitted signal causes the signal to be wider in the frequency domain. Thus, the number of cycles *C* is related to the transmitted bandwidth, so that the lower the number of cycles, the wider the transmitted bandwidth. For instance, if a signal with f=300 kHz and C=4 cycles is transmitted, the 3 dB transmission bandwidth ranges from 252 kHz to 345 kHz. This must be taken into consideration, since the phase velocity at these frequencies might not vary linearly, causing dispersion in the wave packet.

Therefore, once the envelope is considered, the transmitted wave packet p(x,t) will be expressed using Equation (19).

(19) $$p\left(x,t\right)=s\left(x,t\right)\cdot w\left(\frac{x}{c_{g}}\pm t\right)=\sin\left(2\pi f\left(\frac{x}{c_{p}}\pm t\right)+\phi \right)\cdot \hat{w}\left(\frac{x}{c_{g}}\pm t\right)$$

Please note there that instead of using the transmitted envelope w(t) we are using $\hat{w}\left(t\right)$, which changes its shape in function of the distance due to dispersion effects.

It is necessary to consider that under EMAT technology the excitation signal is generated in a set of *N* loops of a coil, separated by a distance *L*, which will generate the propagation wave y(x,t) using Equation (20).

(20) $$y\left(x,t\right)=\sum_{m=1}^{2N}{\left(-1\right)}^{m}p\left(x-m\cdot L/2,t\right)$$

Please note here that each loop generates two signals (one per meander), and that the sign of their contribution to the propagation wave y(x,t) is included in the term ${\left(-1\right)}^{m}$. Besides, the measure is sensed at a distance *D*, in another set of *N* loops separated by a distance *L*. Therefore, the received signal z(t) will be expressed using Equation (21).

(21) $$z\left(t\right)=\sum_{n=1}^{2N}{\left(-1\right)}^{n}y\left(D-n\cdot L/2,t\right)$$

Again, the sign of each meander is represented by the term ${\left(-1\right)}^{n}$. Going back to the previous equations, the total signal sensed from each mode z(t) is obtained.

(22) $$z\left(t\right)=\sum_{m,n=1}^{2N}{\left(-1\right)}^{m+n}p\left(D-\left(m+n\right)\cdot L/2,t\right)$$

(23) $$z\left(t\right)=\sum_{m,n=1}^{2N}{\left(-1\right)}^{m+n}\sin\left(2\pi f\left(\frac{D-(m+n)\cdot L/2}{c_{p}}\pm t\right)+\phi \right)\cdot \hat{w}\left(\frac{D-(m+n)\cdot L/2}{c_{g}}\pm t\right)$$

The signal received from each mode z(t) has different values of $c_{p}$ and $c_{g}$, as it was concluded from Figure 2. Thus, each mode arrives at the receiver with different amplitude and envelope, depending on the attenuation of each mode and the difference of phase when the signal is received in the coil. Therefore, the amount of energy of the received signal can vary at different frequencies.

**Figure 3:**
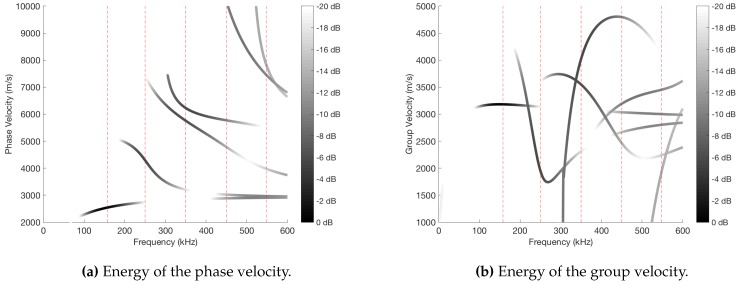
Phase velocity and group velocity of the different modes represented according to the energy in a range of frequencies.

**Figure 4:**
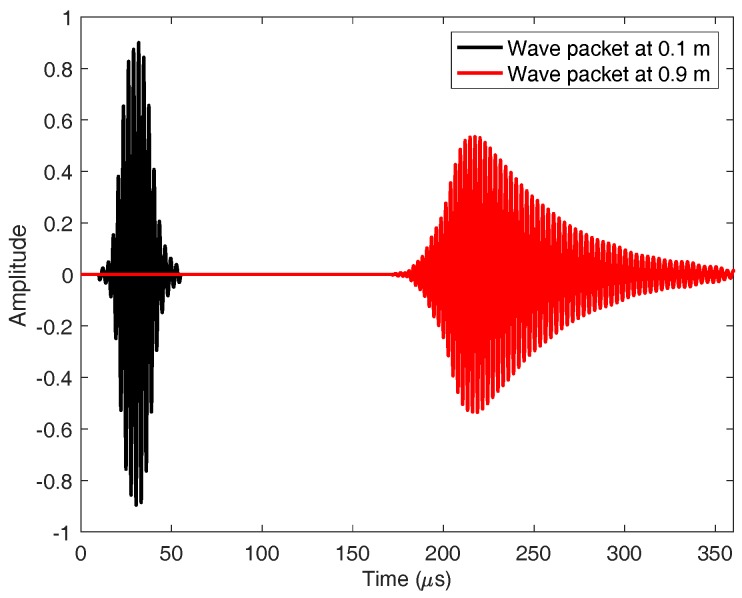
Dispersion suffered by the wave packet when traveling 0.8 m in the pipe (S0 mode, f=300 kHz, C=4 cycles).

In order to find out more about the behavior of the modes in a set frequency range, a frequency sweep was made between 0 and 600 kHz with one coil and C=4 cycles per wave packet. Figure 3 shows the phase velocity (Figure 3a) and group velocity (Figure 3b), where black color means the energy is maximum at that frequency. It is important to indicate that dispersion has been taken into account to carry out the experiments, since the signal has been decomposed with the envelope window $\hat{w}\left(t\right)$ through the Fourier Transform. Thus, the velocities and delays of the different frequencies which are part of the same pulse have been considered. As an example of the effects of dispersion over the wave packet, Figure 4 shows the dispersion suffered by the wave packet when traveling 0.8 m in the pipe (S0 mode, f=300 kHz, C=4 cycles).

The coil used in the experiments has the following parameters: distance between loops L=16.26 mm and N=3 loops. It can be observed in the graphs that the maximum energy appears in f=158 kHz and mode A0. However, there exists a certain periodicity in the energy of the received signals. It implies that the same coil could be used to excite other frequencies, even if it has been designed to get the maximum energy in a set frequency. In fact, different frequencies will be excited in the experiments. Specifically, the frequencies indicated with red dashed lines in Figure 3 will be used, because the energy and the excited modes are different in each of them.

### 2.2. Hardware Description

The technology (Innerspec PowerBox H and the MRUT PMX scanner) and the pipe mock-ups needed to perform the empirical validation of the modeling results were provided by Innerspec Technologies S.L [21], a company which provides NDT solutions using EMAT technology.

The inspection instrument used is the Innerspec PowerBox H, a hand-held battery operated instrument. It is designed for ultrasonic applications that require very high voltages and/or long bursts of energy such as non-contact techniques (EMAT, Air-Coupled) and inspection of highly-attenuating materials. The instrument is capable of generating up to 1200V or 8kW of peak power at speeds of up to 300 Hz.

Guided waves can be used to cover distances ranging from a few millimeters to tens of meters. The two most common techniques for in-service inspections with guided waves are Long Range UT (LRUT) and Medium Range UT (MRUT). All the results showcased within this manuscript were obtained with the MRUT PMX scanner, which is used in both attenuation and reflection mode to cover shorter distances (0.1–5 m). The sensors are mounted on scanners to inspect long stretches of pipes or tanks. It typically works with frequencies from 100 kHz to 1 MHz, and can detect small pits (×10 more sensitivity than using LRUT).

The MRUT PMX scanner allows to scan axially with a single or double sensor on the pipe to measure attenuation and/or velocity changes in the signal due to corrosion, cracks or other defects around the circumference of the pipe. It is ideal for quick inspections of exposed pipe at speeds up to 150 mm/s (6 in/s).

**Figure 5:**
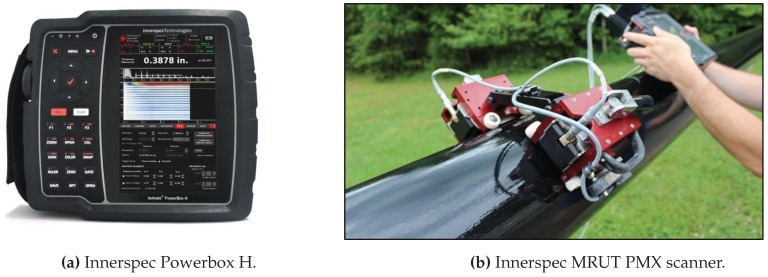
Measuring equipment used in the experiments.

Figure 5 shows the hardware equipment used in the manuscript.

## 3. Effects of the Defect Over the Lamb Waves

The modeling of the pipe by means of the ultrasonic waves is a non-trivial problem. The changing shape of the defects makes difficult to draw general conclusions about the relation between the defect and the received signals. The distortion caused by the defects over the different modes strongly varies with the shape of the defect [12,13]. For instance, the amplitude of the signal, the time of arrival (group velocity $c_{g}$) and the phase velocity $c_{p}$ of the wrap-around signal vary with the dimensions of the defect.

**Figure 6:**
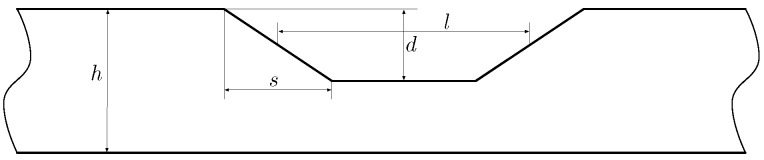
Model of the simulated defects.

To study the relation between these parameters and the shape of the defects, the Finite Element Method (FEM) included in the Partial Differential Equations Toolbox of *Matlab* has been used. A database of 418 defects has been generated using this simulation tool. The defects have been characterized with three parameters: length (*l*), depth (*d*) and slope (*s*). Figure 6 depicts the defect dimensions using the three aforementioned parameters. If s>l the defect is discarded. In the case studied, the thickness of the pipe is z=9.27 mm. Table 1 shows the range of values that the parameters can take.

Likewise, the simulated coil has the following parameters: distance between loops L=16.26 mm, N=3 loops and distance to the receiver D=0.7 m. Besides, each pulse of the signal contains 4 cycles in all the experiments.

**Figure 7:**
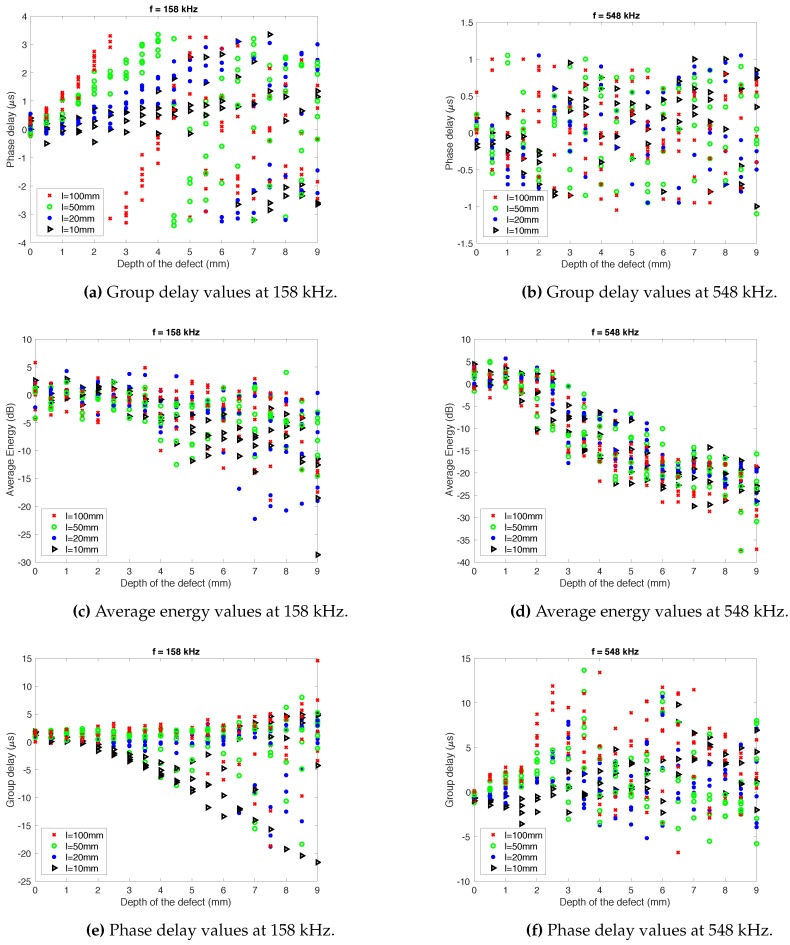
Feature values depending on the depth of the defect.

Figure 7 shows how the depth of the defect affects to the values of group delay (Figure 7a,b), average energy (Figure 7c,d) and phase delay (Figure 7e,f), at two frequencies: f={158,548} kHz. For simplicity, the width of the defect was not modeled, that is to say, that dimension of the pipe was not considered. Each case has a few points since defects with different slope have been considered.

As it can be shown in the graphs, there exists a tendency in some of the considered features. Focusing on the average energy, calculated in f=548 kHz, it is clear to see that, as the length of the defect increases, the average energy decreases drastically, especially between 2 and 6 mm of defect depth. This is exactly what would be expected when there exists a leak in the pipeline and the energy of the signal is scattered through it.

In the case of the group delay, considering the measurement taken at f=158 kHz, it can be observed that the signal tends to be delayed (positive delay) when the defect increases. This does not happen when the length of the defect is very small (*l* = 10–20 mm), since the signal arrives earlier than in the non-defect case. In any event, the aim of this modeling work was to evaluate whether these features contain useful information to tackle the problem addressed.

There exists a difficulty of reaching a conclusion about the relation between the calculated features and the profile of the pipe. Because of that, it is necessary to apply advanced techniques which bring more information about what are the best features or how they should be mixed.

## 4. Smart Sound Processing (SSP) for Defect Sizing

**Figure 8:**
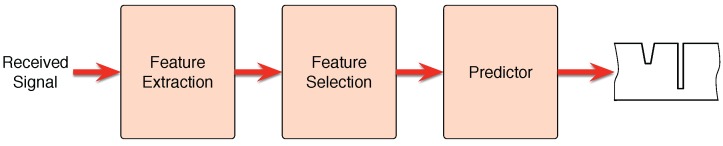
Scheme of an SSP system.

It is necessary to apply SSP methods to solve the defect sizing problem in pipes that is being coped in the current manuscript. This type of methods usually follows the process described in Figure 8. First of all, it is important to extract useful information from the signals on the form of features. Once this is done, the next step is to select the ones that best work to solve the problem at hand. Finally, a predictor will construct a model capable of predicting the solution in an unknown case, such as a new pipeline.

It is important to extract useful information from the received signal in order to be capable of detecting and sizing defects present in the pipes under inspection. With this purpose, different features were elicited:

- Maximum Amplitude (dB). This measure indicates the value of the maximum peak received from the signal. It is determined by looking for the value of the maximum peak around the expected point, which is the position of the maximum of the reference signal form in case of absence of any defects, $t_{0}$).
- Phase Delay (μs). This measure represents the time taken between the pulse shipment and its reception at the same pipe location. It is determined measuring the time difference between the position of the maximum of the reference signal (signal without defect) $t_{0}$ and its closest maximum in the sensed signal. There may be considerable uncertainty in this feature if the delay is higher than half the period, since the nearest peak becomes the maximum of the signal.
- Average Energy (dB). It represents the average energy of the pulse. The interval considered for its calculation started 30 μs before $t_{0}$ and ends up 30 μs after it.
- Group Delay (μs). In order to calculate this measure, the centroid of the average energy of the pulse has been considered. It has been estimated using the centroid of the pulse around the expected maximum ($\hat{t_{g}}$), with Equation (24).
  (24) $$\hat{t_{g}}=\frac{\sum_{t=t_{0}-3\cdot 10^{-5}}^{t_{0}+3\cdot 10^{-5}}tz{\left(t\right)}^{2}}{\sum_{t=t_{0}-3\cdot 10^{-5}}^{t_{0}+3\cdot 10^{-5}}z{\left(t\right)}^{2}}$$

All these features have been extracted directly from the received signal at D=0.7 m. Two additional features, extracted from the reflected signal, where included to study the importance of the analysis of the echos in the sizing problem.

- Maximum Amplitude of the echo (dB).
- Average Energy of the echoes (dB).

It is remarkable the fact that the reflected echoes are not always depicted in the gathered signals, since the position of their contribution depends on the relative position of the defect in the pipeline with respect to the EMAT actuator. Therefore, in some cases the echo is overlapped with the transmitted signal, and cannot be clearly identified. In the simulations two scenarios would be modeled: on the one hand, the case where the reflected echoes are present and on the other hand, the case where they are not, to study the importance of these echo dependent features in the performance of the sizing estimator.

In the problem at hand, 5 excitation frequencies were used. In total, 30 features were extracted taking into account that 6 features were obtained for each excitation frequency. Some of them will work better than others, so it is important to select the best ones and reduce the total amount of them, in order to properly estimate the size of the defect.

To select the features which better work in this experiment, a feature selection process was applied through evolutionary techniques. Evolutionary Algorithms (EAs) are inspired in natural evolution laws and allow to find the optimum solution from the solutions (denoted individuals) obtained in previous iterations [22]. In this paper, a tailored EA has been applied, searching for the best subset of features and trying to minimize the Root Mean Square Error (RMSE) of a Least Squares Linear Discriminant (LSLD). The use of more complex prediction methods has been avoided in the feature selection process, since the EA requires training and testing the predictor a large number of times. The considered constrains are to limit the number of frequencies used as well as the number of features selected.

**Figure 9:**
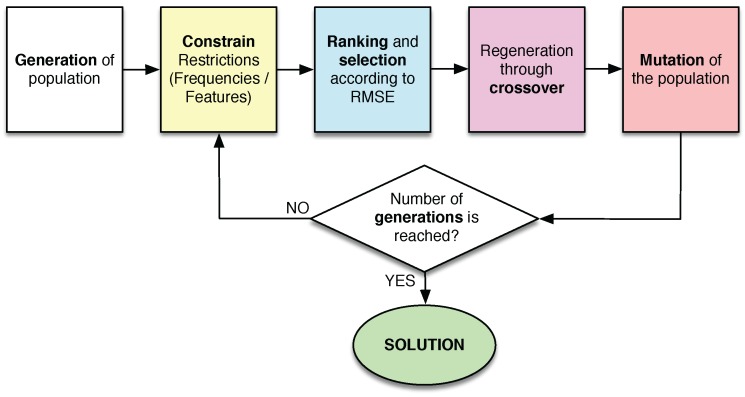
Scheme of the evolutionary algorithm applied in the experiments.

The EA, which is described schematically in Figure 9, is composed of several steps:

1. A population of $N_{p}$ individuals is generated. Each solution consists of a binary vector with a length equal to the total number of features. Thus, ones indicate the features which are selected in the individual, while zeros indicate the features which remain outside.
2. The candidates of the population are restricted to the considered constrains. All of them are modified in order to randomly change the value of some bits until one or the two constrains are fulfilled, depending on the case.
3. A LSLD is designed with the subset of features of each candidate solution. The RMSE of the defect depth is calculated, which is the fitness function in this experiment. With this value, the population is ranked, keeping the best individuals in the top of the ranking.
4. After that, a selection process is applied, which consists in keeping the best 10% of the solutions, removing the remaining ones.
5. The removed solutions (90% of the population) are regenerated by crossover between the best candidates.
6. Mutations are applied to the new population. With this step, 1% of the bits is changed. Furthermore, this step is not applied to the best solution in order to ensure the convergence of the algorithm.
7. This process is repeated from step 2 during $N_{g}$ generations. The final best solution will be the best solution obtained in the last iteration.

Sometimes the EAs do not reach a high convergency. In order to improve it, an elimination tournament of small EAs has been implemented. It consists in joining the winners of the EAs in pairs during several rounds ($N_{r}=6$ in our case), until the best individual reaches the end and, consequently, it becomes the best solution to the problem. 32 small EAs were considered with a population of 100 individuals and 8 generations each, except for the last EA, where 16 generations are configured for convergency [22].

Once the best features have been selected for this specific problem, a non-linear predictor needs to be applied to get the final profile of the pipeline and to know the performance of the developed model. Neural Networks were applied, specifically the Multi Layer Perceptron (MLP) [23]. A perceptron is a neuron with a set of adjustable weights and an activation function by steps [24]. Levenberg-Marquardt algorithm, a method where the minimization function is a sum of quadratic terms [25], was applied for training purposes. The number of hidden neurons was a parameter in the experiments.

A *k*-fold cross validation has been applied in the generated database, being k=12 [26]. This method allows to divide the database in *k* groups so that the full process is repeated *k* times, using one group as test subset and the remaining k−1 groups as training subset. The advantage of this method is that the obtained results are more generalizable. It has been applied in both the feature selection process and the training of the neural network-based predictor.

## 5. Results

The research work showcased in this manuscript was carried out using both real and simulated measurements. This section of the paper will be opened discussing the results obtained during the simulation experiments.

### 5.1. Defect Sizing in Simulated Pipelines

An experiment was developed using the synthetic database described in Table 1. In this case, the RMSE was computed to evaluate the performance of the predictors. The objective is to know how well the received signal is estimated at different frequencies. The *k*-fold cross validation described in Section 4 has been applied in this experiment, so all the results presented here have been obtained with this method.

It is important to indicate that noise from the real measurements was introduced to the signal, the amplitude and the temporal resolution in order to make the experiments as real as possible. A real pipe free from defects was taken in order to study the measuring system tolerance to noise. It was obtained an SNR of 32.93 dB, a variation in time with an standard deviation of 2.1434 samples at 10 MHz (0.2 μs) and a variation in scale of 0.80 dB.

**Figure 10:**
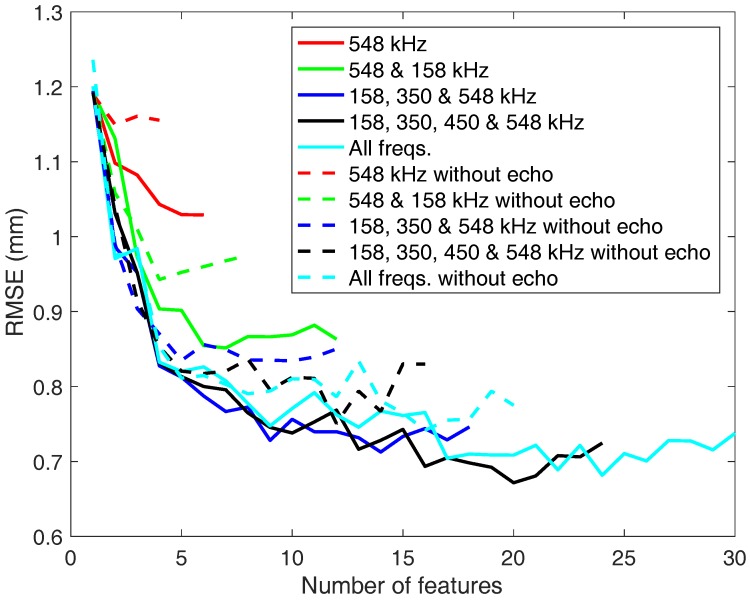
RMSE obtained with neural networks predictor depending on the number of features selected at different frequencies.

As stated above, the relevance of extracting features from echoes related to reflections has been also studied. Figure 10 shows the RMSE obtained by the different predictors, depending on the number of selected features. The different curves show the performance depending on the combination of frequencies considered, in the case of only using features from the wrap-around echo, and in the case of including features from the reflected echo. The combination of frequencies and the classifier used in each case are those that provide the best results in terms of RMSE. It can be seen that the results improve (less RMSE) as the number of evaluated frequencies is increased. It happened especially until 158, 350 and 548 kHz frequencies were evaluated. The results slightly improve when the number of frequencies studied is increased, so there would be no need to make the system more complex to reduce a few millimeters the final rate.

In terms of the reflected echo features, if they are not used (dashed lines) the tendency of the graphs is similar to the previous one. In general, the results are not significantly different from those using echo features, especially when all frequencies are used. Because of that, the features extracted from the echo signals are not essential to evaluate the problem and it is possible to get a good result in case the return of the signal cannot be evaluated.

**Figure 11:**
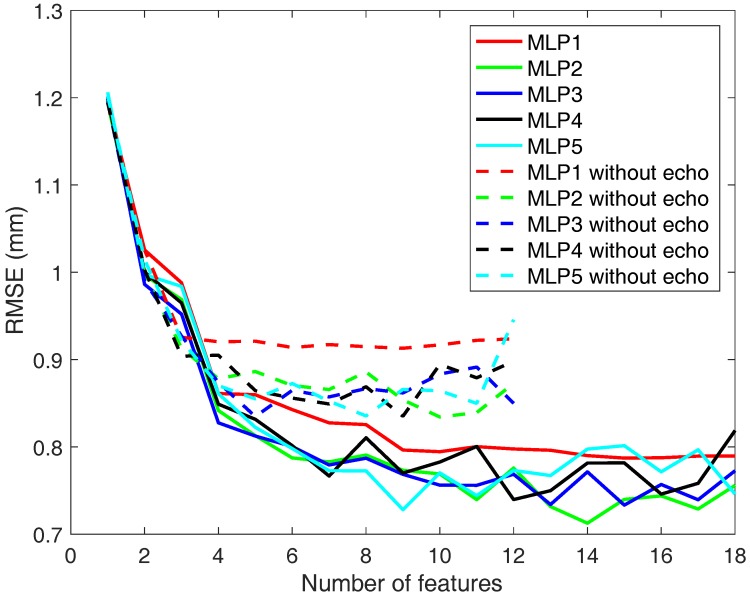
RMSE obtained depending on the number of selected features considering different number of neurons in the MLPs.

With regard to the used predictors, Figure 11 shows the RMSE depending on the number of features employed and the number of neurons configured in the MLP (1, 2, 3, 4 and 5). Although the results are good using just one neuron, they can be improved by using two. However, if the number of neurons is further increased (3–5), the result is not much better than before.

In all the cases above it is clear that as the number of features is higher, the results improve. It happens especially in the range between 1 and 5 features, where the error falls considerably in Figure 10 and Figure 11.

Now the features selected at different frequencies from the proposed ones are going to be studied. With this purpose the manuscript will focus on the case of mixing eight features from three frequencies: 158, 350 and 548 kHz (dark blue line). The results do not improve substantially when more frequencies are added or when the number of features is increased. In Table 2 and Table 3 the ratios of selection of the features are shown. The first one includes the case of considering the reflected echo features and the second the case of not considering them. The RMSE associated to these two cases are 0.79 mm and 0.87 mm, with and without reflected echo features, respectively.

The results show that the most important feature is the average energy of the received pulse of the signal, since it is selected in all the frequencies considered. It confirms that the energy is the most affected parameter when the signal travels through a defect. This reasoning applies for both the wrap around echo and the reflected echo.

Other features which work properly are the maximum amplitude and the group delay, but mainly when the features extracted from the reflected echo are not taken into consideration. It means that they are good features, but not as much as the energy from the reflected echo. Furthermore, features like phase delay are not relevant for the study, because they are not selected to get a better result. In fact, this feature has 0% of selection in all cases of study. This is caused by the problems with the uncertainty in measuring this parameter.

These results demonstrate again that the reflected echo features are not essential for the problem. Using them the model fits just 0.1 mm better with the target.

### 5.2. Defect Sizing in a Real Pipeline

**Figure 12:**
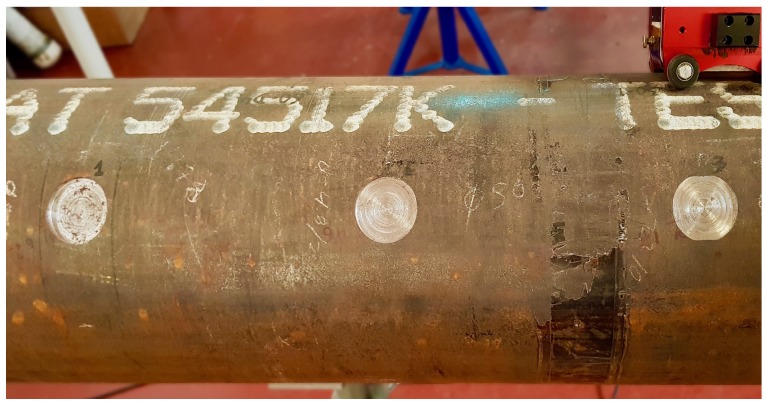
Image of the real pipe.

Now the results obtained during the experimental trials in a pipe mock-up are presented. Data has been collected using MRUT PMX scanner on a pipe, as mentioned before. The inspection was performed moving the sensor axially (sending the waves circumferentially) on a pipe which includes three flat defects with different depths. An image of the pipe is shown in Figure 12, while its specifications are shown in Table 4.

**Figure 13:**
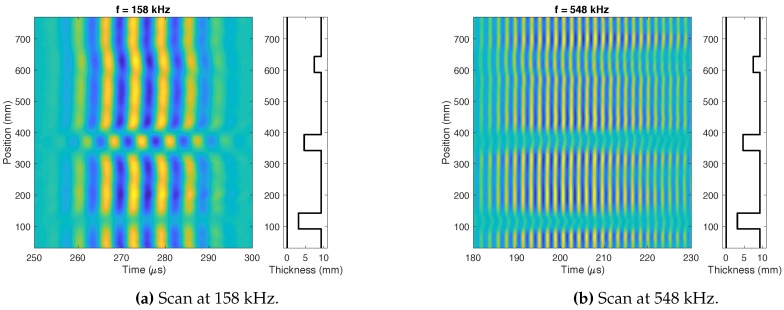
Axial scan of the real pipeline at two frequencies.

The inspections were carried out at different frequencies. The distortions introduced by the defect over the analyzed signals are shown in Figure 13. Axial scans sending the waves circumferentially have been carried out every millimeter of the pipeline, and the main wave packages of the wrap-around echos have been stored. Different behaviors are depicted depending on the depth of the defects and the excited frequency, *f* = 158 kHz (Figure 13a) and *f* = 548 kHz (Figure 13b).

The graphs included in Figure 13 show that the defects change the amplitude and the phase of the received signals. Furthermore, these distortions are different depending on the size of the defects and the excited frequency *f*.

The profile of the real pipe will be modeled using the features explained above. In this experiment, the predictor trained was used along with the synthetic database and it was also tested with the signals sensed in the real laboratory trials. Therefore, it has not been necessary to apply the cross validation technique, as the training and test subsets were clearly defined. Three cases were developed: considering 4 features at 158 kHz, 4 features at 548 kHz and mixing all of them. The 2 features from the reflected echo (maximum amplitude of the reflected echo and average energy of the reflected echo) have not been considered because the return from the signal was sometimes overlapped with the excitation pulse, so it was not posible to extract any information from it.

**Figure 14:**
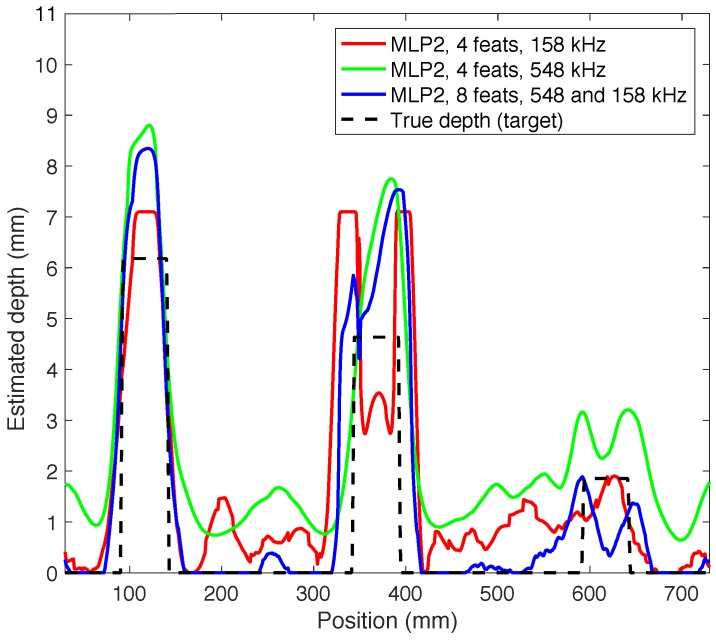
Estimation of the real pipeline model.

In Figure 14 and Table 5 the results of the described experiments are shown. The model that better fits with the pipeline is the predictor which considers eight features and the two frequencies. It is the one which better distinguishes between defect and non-defect areas, since the estimation of the defect depth is very close to zero in non-defect areas. In fact, the RMSE is the best of the three predictors (1.48 mm). When mixing the information from the two frequencies the results are quite improved. With the other models the estimation was not so good, especially when there were no defects in the profile.

## 6. Discussion

In this work the defect detection and sizing in steel pipelines have been studied. With this purpose, Lamb waves have been generated with EMAT-based techniques. These have been analyzed with SSP methods, in order to try to model the pipeline. After the experiments described above have been carried out, the following conclusions have been drawn:

- The shape of the defect causes differences in the received signal. It is not feasible to obtain an analytical solution for all the cases.
- The extracted features are useful for the pipeline sizing problem, since the results are good in terms of RMSE. The average energy and the maximum amplitude from the signals are particularly relevant for the study.
- It is important to excite the waves at several frequencies because the behavior and velocity of the Lamb modes is totally different depending on this parameter.
- Related to the predictors, a large number of neurons in the MLPs is not required. When it is increased above two or three neurons, the results do not improve significantly.

In the future it would be possible to increase the amount and variety of defects in real pipelines. Besides, more features could be applied, such as some from the signal in successive wrap-arounds.

## 7. Conclusions

Pipeline inspection problem can be approached in many different ways. Lamb wave generation through EMAT actuators proves to be a very effective and useful one. However, the amount of information provided by the wrap-around signals needs to be processed by advanced techniques, such as smart sound processing algorithms. Thanks to them, it is feasible to get good estimation results of the pipeline defects, in both real and simulated signals. In the manuscript it has been demonstrated the importance of applying a multi-frequency study for defect sizing problem, the relevance of some features from the signals (e.g., energy and amplitude) and the absence of the need to greatly increase the complexity of the classifiers to get a good estimation in the problem at hand.

## Figures and Tables

**Table 1:** Range of values for the different parameters of the defects.

Parameter	Number of Possible Values	Values
Frequency (kHz)	5	158, 250, 350, 450, 548
Length (mm)	4	10, 20, 50, 100
Depth (mm)	19	0, 0.5, 1,..., 9
Slope (mm)	7	1, 3, 5, 10, 50, 100

**Table 2:** Ratios of selection of the features, including echo features.

Feature	Frequency (kHz)		
	158	350	548
Maximum Amplitude	0%	92%	0%
Phase Delay	0%	0%	0%
Average Energy	100%	100%	100%
Group Delay	0%	0%	58%
Maximum Amplitude of Echo	8%	50%	0%
Average Energy of Echos	92%	100%	100%

**Table 3:** Ratios of selection of the features without echo features.

Feature	Frequency (kHz)		
	158	350	548
Maximum Amplitude	100%	100%	92%
Phase Delay	0%	0%	0%
Average Energy	100%	100%	100%
Group Delay	8%	100%	100%

**Table 4:** Specifications of the real pipe.

Parameter	Value
Material	Structural Steel S355NH
Thickness (mm)	9.27
Depth of Defect 1 (mm)	6.18
Depth of Defect 2 (mm)	4.63
Depth of Defect 3 (mm)	1.85

**Table 5:** Results of real database.

Predictor	Number of feats	Frequency (kHz)	RMSE (mm)
MLP 2 neurons	4	158	1.84
	4	548	1.80
	8	158, 548	1.48
